# Prevalence and risk factors for prolonged QT interval and QT dispersion in patients with type 2 diabetes

**DOI:** 10.1007/s00592-016-0864-y

**Published:** 2016-04-23

**Authors:** Vladan M. Ninkovic, Srdjan M. Ninkovic, Vanja Miloradovic, Dejan Stanojevic, Marijana Babic, Vojislav Giga, Milan Dobric, Michael I. Trenell, Nebojsa Lalic, Petar M. Seferovic, Djordje G. Jakovljevic

**Affiliations:** 1 Department of Cardiology, Specialist Hospital Merkur, Bulevar Srpskih Ratnika 18, 36210 Vrnjacka Banja, Serbia; 2 Clinical Centre, Kragujevac, Serbia; 3 Medical School, University of Kragujevac, Kragujevac, Serbia; 4 Cardiology Department, Clinical Centre of Serbia, Medical School, University of Belgrade, Belgrade, Serbia; 5 Institute of Cellular Medicine, Faculty of Medical Sciences, Medical School, Newcastle University, Framlington Place, William Leech B., NE2 4HH Newcastle upon Tyne, UK; 6 Research Councils UK Centre for Ageing and Vitality, Newcastle University, Newcastle upon Tyne, UK; 7 Clinical Research Facility, Royal Victoria Infirmary, Newcastle upon Tyne, UK

**Keywords:** Type 2 diabetes, ECG, QT interval, QT dispersion

## Abstract

**Aims:**

Prolonged QT interval is associated with cardiac arrhythmias and sudden death. The present study determined the prevalence of prolonged QT interval and QT dispersion and defined their clinical and metabolic predictors in patients with type 2 diabetes.

**Methods:**

Cross-sectional study included 501 patients with type 2 diabetes. A standard 12-lead electrocardiogram was recorded. QT corrected for heart rate (QTc) >440 ms and QT dispersion (QTd) >80 ms were considered abnormally prolonged. QTc ≥ 500 ms was considered a high-risk QTc prolongation. Demographic, clinical and laboratory data were collected. Independent risk factors for prolonged QTc and QTd were assessed using logistic regression analysis.

**Results:**

Prevalence of QTc > 440 ms and QTd > 80 ms were 44.1 and 3.6 %, respectively. Prevalence of high-risk QTc (≥500 ms) was 2 % only. Independent risk factors for QTc prolongation >440 ms were mean blood glucose (*β* = 2.192, *p* < 0.001), treatment with sulphonylurea (*β* = 5.198, *p* = 0.027), female gender (*β* = 8.844, *p* < 0.001), and coronary heart disease (*β* = 8.636, *p* = 0.001). Independent risk factors for QTc ≥ 500 ms were coronary heart disease (*β* = 4.134, *p* < 0.001) and mean blood glucose level (*β* = 1.735, *p* < 0.001). The independent risk factor for prolonged QTd was only coronary heart disease (*β* = 5.354, *p* < 0.001).

**Conclusions:**

Although the prevalence of prolonged QTc > 440 ms is significant, the prevalence of high-risk QTc (≥500 ms) and QTd > 80 ms is very low in patients with type 2 diabetes. Hyperglycaemia and coronary heart disease are strong predictors of high-risk QTc.

## Introduction

The QT interval in the surface electrocardiogram (ECG) reflects the total duration of depolarization and repolarization of the ventricles. QT dispersion (QTd) represents nonuniformity of regional myocardial ventricular repolarization and is reflected by differences of the QT interval duration between ECG leads. Both prolonged QT interval and prolonged QTd form a substrate for malignant ventricular arrhythmias. Previous studies showed that a prolonged QT interval and QT dispersion are predictors of cardiovascular mortality and all-cause mortality in patients after acute myocardial infarction [1], patients with heart failure [2], patients with diabetes type 1 and 2 [3, 4], patients with idiopathic long QT syndrome [5] and in the population of apparently healthy individuals [6, 7].

The aims of this study were to determine the prevalence of prolonged QT interval and QT dispersion in patients with type 2 diabetes and their correlations with clinical and metabolic parameters, with particular emphasis on coronary heart disease, the parameters of glycaemic control and type of diabetes treatment.

## Methods

### Study design and patients

This cross-sectional study included 501 Caucasian patients with type 2 diabetes (277 men) treated at the National Educational Centre and Hospital for Diabetes, “Merkur” Vrnjačka Banja, Serbia, from September 2011 until December 2012. Study was approved by the local research committee and all the procedures were according to Declaration of Helsinki and all study participants provided written informed consent. The mean age (SD) was 60.4 (8.1) years, and the mean duration of diabetes (SD) was 9.9 (6.8) years.

Type 2 diabetes was defined as a diagnosis made after 39 years of life with the lack of need for insulin treatment within the first year after diagnosis. Hypertension was defined as systolic blood pressure >140 mmHg and/or diastolic blood pressure >90 mmHg during ≥2 measurements, or current antihypertensive therapy. Retinopathy was assessed by retinal photographs centrally graded by a single observer. Distal symmetric neuropathy was diagnosed according to the EMNG findings, or relevant signs and symptoms including determination of vibratory perception threshold. Coronary heart disease was defined as the history of previous myocardial infarction, unstable angina, coronary artery bypass grafting, percutaneous coronary intervention, positive findings on coronary angiography, positive ECG stress test, typical effort angina, or Minnesota code 1.1 or 1.2 in a surface ECG [8].

Estimated glomerular filtration rate (eGFR) was calculated according to serum creatinine level using the MDRD formula [9].

All patients were free of clinical signs of the existence of cardiovascular autonomic neuropathy (i.e. orthostatic hypotension). The study excluded patients with ECG signs of myocardial hypertrophy, bundle branch block, as well as patients on treatment with drugs known to affect the QT interval, especially antiarrhythmic class I and III, digitalis, and antidepressants.

### QT interval length and QT dispersion

In a standard ECG (Schiller AT1, Cardiovit, Switzerland), recorded in a supine position, RR and QT intervals were measured by two independent observers, who were blinded to patients’ data, using a ruler and magnifying glass. QT interval was measured from the beginning of QRS complex to the end of the T wave in the intersection with the isoelectric line [10]. QT corrected for the length of the previous cycle (QTc) was obtained using Bazett’s formulae: QTc = QT/√RR (sec) [11].

QTc is the mean of QTc from five consecutive cycles in lead V5. QTc > 440 ms was considered abnormally prolonged, whereas QTc ≥ 500 ms was considered high-risk QT prolongation. RR and QT intervals were also measured in three consecutive cycles in each of the six thoracic lead. QTc dispersion (QTd) was calculated as the difference between the maximum and minimum QTc at any thoracic lead. QTd > 80 ms was considered an abnormally prolonged [12].

For each patient the values of QTc and QTd represent average values of the readings of the two observers. ECG recordings were performed on the same day as daily glycaemia profile.

### Parameters of glycaemic control

After hospitalization, the patients were put on a dietary regimen. For each patient calorie intake was calculated based on ideal body weight (IBW) and estimates of daily calorie consumption (25–30 kcal/IBW). The composition of the meal was carbohydrates 60 %, protein 20 % and fat 20 %. Meals were taken at 7:30 AM, 12:00 AM and 6:00 PM. On the second day of admission, glycaemic daily profile was determined for each patient. Glucose levels were determined from capillary blood sample using the glucose hexokinase assay (EKF Diagnostics, Germany). In patients treated with oral hypoglycaemic agents, daily profile consists of six capillary blood glucose values: three before each main meal (at 7:30 AM, 12:00 AM, and 6:00 PM) and three 2 h after the main meal (at 10:00 AM, 2:30 PM and 8:30 PM). In patients who received insulin, daily glycaemia profile included two additional blood samples taken at 12:00 midnight and 3:00 AM, giving a total of 8 values. From the analysis were excluded patients with hypoglycaemic values in daily glycaemia profile, due to potential influence of reflex sympathetic stimulation on the length of the QT interval. Hypoglycaemia was defined as glucose levels <3.9 mmol/L (70 mg/dL).

The following parameters of glycaemic control were used: fasting blood glucose (FBG), the mean postprandial glucose, the mean blood glucose (MBG), the mean amplitude of glycaemic excursion (MAGE) and glycated haemoglobin A1c (HbA1c). To assess glycaemic fluctuations during the day, we used the MAGE, described by the Service et al. [13]. It is calculated as the arithmetic mean of the absolute differences of the peak and subsequent nadir of blood glucose values in daily glycaemia profile, wherein in calculation are taken only differences >1 SD of the mean glycaemia value. The MAGE is an independent measure of mean blood glucose value designed to evaluate daily fluctuations in blood glucose. A significant relationship was previously demonstrated between such model of discontinuous glucose sampling and model of continuous subcutaneous glucose monitoring [14]. All laboratory data were centrally collected and patients’ screening was performed during hospitalization period with the same instrument and observer.

### Statistical analysis

All statistical analysis was carried out using SPSS version 21.0 (SPSS Inc. Chicago, IL). Prior to statistical analysis, data were checked for univariate and multivariate outliers using standard Z-distribution cut-offs and Mahalanobis distance tests. Normality of distribution was assessed using a Kolmogorov–Smirnov test. Differences in clinical characteristics of patients between subgroups of QTc (≤440 or >440 ms duration) and QTc dispersion (≤80 or >80 ms) were assessed using the *t* test for continuous variables as appropriate; results are shown as mean ± standard deviation (SD). Differences between categorical variables were assessed using Chi-squared test. To assess variables that were independently predictive of prolonged QTc duration and QTc dispersion, we used logistic regression analysis. Variables entered into initial model were age, sex, diabetes duration, body mass index, systolic and diastolic blood pressure, coronary heart disease, stroke, retinopathy, polyneuropathy, diabetes therapy (metformin, sulphonylurea, insulin), fasting glucose, mean postprandial glucose, mean blood glucose, MAGE, HbA1c, total cholesterol, triglycerides, and estimated glomerular filtration rate. Variables were retained in the final model if they added significantly to the likelihood of models or to the estimated coefficients of predictors. Statistical significance was indicated if *p* < 0.05.

## Results

Patients’ demographic, clinical and metabolic data are presented in Table 1. Prevalence of prolonged QTc duration and QTc dispersion in patients with type 2 diabetes were 44.1 and 3.6 %. Additionally, 2 % of patients demonstrated prolonged QTc interval of ≥500 ms. QTc duration and dispersion were affected by the gender with females demonstrating significantly higher prevalence of prolonged QTc duration (both >440 and >500 ms) than men (55 vs 36 %, and 4 vs 1 %, *p* < 0.05) and QTc dispersion (5 vs 3 %, *p* < 0.05; Table 2).

**Table 1:** Patients demographic, clinical and metabolic characteristics

Total number of patients	501
Gender (male/female)	277/224
Age (years)	60.4 ± 8.1
Diabetes duration (years)	9.9 ± 6.9
Body mass index (kg/m^2^)	30.4 ± 4.8
Systolic blood pressure (mmHg)	136.7 ± 48.7
Diastolic blood pressure (mmHg)	80.8 ± 7.0
Coronary heart disease (%)	22.9
Stroke (%)	6.8
Retinopathy (%)	47.5
Polyneuropathy (%)	74.5
Metformin (%)	77.2
Sulphonylurea (%)	38.5
Insulin (%)	69.1
Fasting glucose (mmol/l)	8.9 ± 2.9
Mean postprandial glucose (mmol/l)	10.4 ± 3.5
Mean blood glucose (mmol/l)	9.3 ± 3.0
MAGE (mmol/l)	4.1 ± 2.1
HbA1c (%)	8.1 ± 1.4
Total cholesterol (mmol/l)	5.8 ± 1.5
Triglycerides (mmol/l)	2.1 ± 1.6
eGFR (ml/min/1.73 m^2^)	96.4 ± 22.7

*MAGE* mean amplitude of glycaemic excursion, *eGFR* estimated glomerular filtration rate

**Table 2:** Prevalence of increased QTc interval duration and QTc dispersion in patients with type 2 diabetes

	Males (*n* = 277)	Females (*n* = 224)	Total (*n* = 501)
Increased QTc duration (>440 ms)	35.7 (28.2–39.1)	54.5 (46.0–61.2)*	44.1 (39.6–52.4)
Increased QTc duration (≥500 ms)	0.7 (0.4–1.1)	3.5 (2.7–4.6)*	2.0 (1.6–2.4)
Increased QTc dispersion (>80 ms)	2.9 (2.3–3.3)	4.5 (3.7–5.1)*	3.6 (3.0–4.1)

Values are given as % (95 % CI)

*QTc* QT interval corrected for heart rate, *CI* confidence interval

* Significantly higher than Males (*p* < 0.05)

Patients with prolonged QTc interval had significantly higher age (*p* < 0.02), body mass index (*p* < 0.01), and prevalence of coronary heart disease (*p* < 0.01), retinopathy (*p* < 0.01), polyneuropathy (*p* < 0.01), and the use of sulphonylurea (*p* < 0.01; Table 3). Measures of metabolic control, i.e. HbA1c, daily glucose fluctuation (MAGE), mean blood glucose, fasting blood glucose, mean postprandial glucose and triglycerides, were significantly higher in patients with QTc interval duration of >440 ms (*p* < 0.05; Table 3). Patients with prolonged QTd showed significantly higher age and prevalence of coronary artery disease.

**Table 3:** Demographic, clinical and metabolic characteristics of patients with type 2 diabetes by QTc duration and QTc dispersion

	QTc duration			QTc dispersion		
	<440 ms	>440 ms	*p*	<80 ms	>80 ms	*p*
	(*n* = 280)	(*n* = 221)		(*n* = 483)	(*n* = 18)	
Age (years)	59.6 ± 8.0	61.4 ± 8.1	**0.017**	60.2 ± 8.1	64.8 ± 7.7	**0.023**
Diabetes duration (years)	9.5 ± 6.8	10.3 ± 6.7	0.205	9.8 ± 6.5	11.5 ± 6.4	0.298
Body mass index (kg/m^2^)	29.9 ± 4.5	31.1 ± 5.2	**0.005**	30.4 ± 4.9	30.3 ± 3.7	0.921
Systolic blood pressure (mmHg)	135.9 ± 63.2	137.6 ± 17.3	0.665	136.8 ± 49.5	133.6 ± 17.1	0.497
Diastolic blood pressure (mmHg)	80.4 ± 6.5	81.4 ± 7.6	0.140	80.9 ± 7.0	79.4 ± 6.6	0.379
Coronary heart disease (%)	15.4	33.1	**<0.001**	21.3	66.7	**0.001**
Stroke (%)	5.1	7.7	0.520	5.6	5.3	0.969
Retinopathy (%)	35.4	57.6	**0.002**	32.5	38.4	0.815
Polyneuropathy (%)	68.6	80.1	**0.005**	72.7	77.5	0.699
Metformin (%)	77.5	76.9	0.879	77.2	77.9	0.985
Sulphonylurea (%)	33.2	45.2	**0.006**	38.3	44.9	0.622
Insulin (%)	71.1	66.5	0.277	68.9	72.4	0.770
Fasting glucose (mmol/l)	8.3 ± 2.3	9.6 ± 3.3	**<0.001**	8.9 ± 2.9	8.3 ± 2.1	0.305
Mean postprandial glucose (mmol/l)	9.5 ± 2.7	11.5 ± 4.1	**<0.001**	10.4 ± 3.5	10.5 ± 2.9	0.814
Mean blood glucose (mmol/l)	8.5 ± 2.2	10.3 ± 3.6	**<0.001**	9.2 ± 3.1	9.5 ± 2.7	0.741
MAGE (mmol/l)	3.8 ± 1.8	4.4 ± 2.3	**0.004**	4.1 ± 2.0	4.9 ± 2.8	0.267
HbA1c (%)	7.9 ± 1.3	8.3 ± 1.4	**0.001**	8.1 ± 1.4	8.0 ± 1.0	0.822
Total cholesterol (mmol/l)	5.6 ± 1.6	5.7 ± 1.4	0.706	5.7 ± 1.5	4.9 ± 1.1	0.380
Triglycerides (mmol/l)	2.0 ± 1.0	2.3 ± 2.1	**0.022**	2.1 ± 1.6	2.0 ± 0.7	0.605
eGFR (ml/min/1.73 m^2^)	94.9 ± 24.6	98.2 ± 30.9	0.461	97.1 ± 20.0	75.7 ± 44.5	0.346

*p* < 0.05 denotes statistical significance

*MAGE* mean amplitude of glycaemic excursion, *eGFR* estimated glomerular filtration rate

There was a significant moderate relationship between the QTc duration and QTc dispersion (*r* = 0.36, *p* < 0.001). However, 50 % of patients with a normal QT interval dispersion (<80 ms) showed an increased QTc interval duration (>440 ms).

In multivariate logistic regression analyses and after adjustment for age and gender, the independent predictors of prolonged QTc interval were mean blood glucose (*β* = 2.192, *p* < 0.001), female gender (*β* = 8.844, *p* < 0.001), coronary artery disease (*β* = 8.636, *p* = 0.001), and treatment with sulphonylurea (*β* = 5.198, *p* = 0.027; Table 4). On the other note, only coronary heart disease was independent predictor a prolonged QTc dispersion (*β* = 5.354, *p* < 0.001), adjusted for age and gender. Finally, the independent predictors of increased QTc duration of ≥500 ms were coronary heart disease (*β* = 4.134, *p* < 0.001) and mean blood glucose level (*β* = 1.735, *p* < 0.001).

**Table 4:** Predictors of prolonged QTc interval and QTc dispersion in patients with type 2 diabetes

	*β*	*p*
*QTc duration* > *440* *ms*		
Mean blood glucose	2.192	<0.001
Coronary artery disease	8.636	<0.001
Treatment with sulphonylurea	5.198	0.027
Female gender	8.844	<0.001
*QTc duration* > *550* *ms*		
Mean blood glucose	1.736	<0.001
Coronary artery disease	4.134	<0.001
*QTc dispersion*		
Coronary artery disease	5.354	<0.001

## Discussion

In our population of patients with type 2 diabetes, the prevalence of QTc > 440 ms is relatively high and accounts to 44.1 %. However, the prevalence of high-risk QTc ≥ 500 ms is only 2 %. It has been shown in patients with congenital and acquired long QT syndrome that malignant arrhythmias are most often associated with values of 500 ms or more [15–18]. Our data show that the largest proportion of patients with prolonged QTc is in the “grey zone” between arbitrarily taken value of 440 and 500 ms, and that the percentage of patients with a real risk of malignant arrhythmias is low (2 %). The prevalence of pathological QTd (>80 ms) is also low (3.6 %).

These findings are important as they demonstrate that the prevalence of highly prolonged QTc and QTd, which can be associated with severe arrhythmias, is low in patients with type 2 diabetes. From our clinical practice we know that sudden death due to life-threatening arrhythmias in patients with type 2 diabetes is not very common. Sudden death is rather caused by acute cardiovascular events, i.e. myocardial infarction and stroke. Coronary artery disease and poor metabolic control are often the triggers of high-risk QTc and sudden death.

Previous studies in patients with type 2 diabetes showed that the prevalence of QTc and QTd is markedly different and ranged from 15.4 to 67 % for QTc, and from none to 33 % for QTd [19–22]. Such large differences may result from inaccurate identification of the beginning and the end of the QT interval by the observer or the software in the case of automatic analysis of ECG tracings, or the result of selection bias. Furthermore, QTd differences are consequence of different cut-off values in the definition of pathological QTd (between 50 and 80 ms).

There are several risk factors connected with repolarization disturbances in previous studies. Earlier studies have suggested a relationship between diabetic cardiovascular autonomic neuropathy (CAN) and QT prolongation. Since the determination of CAN using classical cardiovascular tests is complicated [23], it has been proposed that determination of QTc duration is specific and easier methods for detection of CAN [24]. However, later studies have not confirmed association of prolonged QT and CAN in diabetes type 1 and 2 [25, 26]. Moreover, a large meta-analysis that investigated the sensitivity and specificity of prolonged QT interval for the detection of CAN in patients with diabetes suggested that prolonged QT interval is a poor indicator of CAN [27]. Previous studies have also not demonstrated significant relationship between QTd and autonomic dysfunction in diabetic patients [28].

Several other risk factors were indicated in prolonged QT interval in diabetes, including systolic and diastolic blood pressure [22, 29, 30], hyperglycaemia [22, 31], serum insulin level [29, 32], microvascular diabetic complication [22, 33] and coronary heart disease [20, 29]. As risk factors for prolonged QTd in the literature are also reported CAN [34], arterial hypertension [28], and coronary heart disease [20, 35]. It has become clear that independent risk factors for QTc and QTd prolongation in patients with type 2 diabetes are different and depend primarily on the set of variables tested.

Independent risk factors for QTc > 440 ms in patients with type 2 diabetes in our study group were: hyperglycaemia, the use of sulphonylurea agents in the treatment of diabetes, female gender and coronary heart disease. Independent risk factors for QTc ≥ 500 ms were only hyperglycaemia and coronary heart disease. Independent risk factor for QTd > 80 ms was coronary heart disease.

It was previously suggested that hyperglycaemia may lead to QT prolongation through several mechanisms including stimulation of protein kinase C leading to reduced synthesis and release of endothelial derived nitric oxide [36, 37]. This consequently lead to decrease in activity of Na+ K+ ATPase, an enzyme responsible for the maintenance of basal membrane potential of myocytes, and cells of the conduction system (by maintaining a high concentration of intracellular K and high concentration of extracellular Na by mechanism of active transport) [36, 37]. Reduced nitric oxide bioavailability during hyperglycaemia is probably responsible for decreased activity of Ca^2+^ ATPase as well, an enzyme in the membrane of myocytes that through active transport mechanism maintains a low concentration of Ca^2+^ ions in the cell, resulting in an increase in the influx of Ca^2+^ ions during phase 2 of the action potential and extension of QT interval [38].

Correlation between hyperglycaemia and QT interval has been demonstrated in previous clinical studies including patients with impaired glucose metabolism and in apparently healthy individuals [22, 31, 39, 40]. Moreover, in both clinical studies and experimental models, it has been suggested that acute hyperglycaemia leads to prolonged QT interval not only in healthy subjects but also in those with diabetes [41, 42].

Our results are in agreement with these suggestions, given that the mean blood glucose derived from daily glycaemia profile is independent risk factor for prolonged QT interval, with a note that the daily profile of glycaemia was determined the same day as the ECG recordings. On the other hand, the relationship between glycaemia and QTd has not been previously reported [28].

The effect of sulfonylurea agents on the incidence of cardiovascular events in patients with diabetes is still controversial. The closure of KATP channels in myocytes under the influence of sulfonylurea leads to the depolarization of the plasma cell membrane, opening of voltage-dependent Ca^2+^ channels with an increase in the influx of Ca^2+^ ions into the cell which generates a prolongation of action potential during phase 2 of the action potential or plateau phase. This results in a prolongation of the QT interval in the ECG. The data in the literature on the relationship between the treatment of diabetes with sulfonylureas and QT prolongation are scarce. In a small group of subjects, Najeed and collaborators have found a significant increase in QTc interval (and QT dispersion) after 2 months of treatment with glibenclamide (from 433 ± 24 to 467 ± 24 ms), as opposed to patients receiving treatment with metformin [43].

Influence of female gender on QT prolongation is well established [33]. Normal adult women have longer QT intervals than men, so widely accepted cut-off value for the normal QTc interval is 440 ms for men and 460 ms for women [44]. Moreover, as to heart rate dependence of the QT interval, adult women have longer QT intervals at longer cycle lengths than men [45]. However, electrophysiological substrate for such gender differences is largely unknown.

During ischaemia, sympathetic hyperactivity accompanied by an underlying myocardial structural damage is likely to increase the ventricular repolarization duration measured as QT interval on the body surface electrocardiogram. There is strong evidence suggesting the association between QT abnormalities and acute and chronic cardiac ischaemia [46, 47]. Moreover, QT prolongation and QTd can be reduced by successful revascularization [48].

The underlying mechanism during acute ischaemia responsible for the heterogeneity of repolarization (QTd) could be identified on a cellular level as a decrease of the resting membrane potential in the course of acute ischaemia, leading to a cellular uncoupling and a shortening of the action potential duration [49].

In chronic ischaemic heart disease, QTd seems to represent the sum of several adverse cardiac abnormalities such as fibrosis, hypertrophy, dilatation, ischaemia and probably, autonomic dysfunction [50]. Obviously, QTd could be considered as a non-invasive marker of potentially lethal underlying cardiac abnormalities—the most important being ischaemia. Our results are in accordance with the findings of these studies and studies that have investigated the association between cardiac ischaemia and repolarization disturbances in patients with type 2 diabetes [20, 29, 35].

### Study limitations

The present study is not without limitations. The lack of longitudinal data and follow-up patients’ clinical course prevents generalization of the data and identification of predictors associated with increased morbidity and mortality in patients with type 2 diabetes, and in particular development of potential life-threatening arrhythmias in those with prolonged QTc interval. Only Caucasian patients were included and prevalence and risk factors for prolonged QTc interval and QT dispersion in patients with other ethnic origins remains to be defined.

## Conclusion

The prevalence of QT prolongation >440 ms is relatively high (44.1 %), but the prevalence of high-risk QT prolongation (≥500 ms), and QT dispersion >80 ms in patients with type 2 diabetes is low (2 and 3.6 % respectively). The use of sulfonylurea agents in the treatment of diabetes and female gender are reasons for clinically irrelevant QT prolongation in patients with type 2 diabetes. Risk factors for high-risk prolonged QT interval (≥500 ms) are acute hyperglycaemia and history of coronary heart disease. The independent risk factor for prolonged QT dispersion in type 2 diabetes is coronary heart disease.
